# Patient-derived xenograft models of breast cancer and their predictive power

**DOI:** 10.1186/s13058-015-0523-1

**Published:** 2015-02-10

**Authors:** James R Whittle, Michael T Lewis, Geoffrey J Lindeman, Jane E Visvader

**Affiliations:** Department of Medical Oncology, The Royal Melbourne Hospital, Grattan St, Parkville, VIC 3050 Australia; Department of Molecular and Cellular Biology, Lester and Sue Smith Breast Center, Baylor College of Medicine, Houston, TX 77030 USA; Department of Radiology, Lester and Sue Smith Breast Center, Baylor College of Medicine, Houston, TX 77030 USA; ACRF Stem Cells and Cancer Division, The Walter and Eliza Hall Institute of Medical Research, Parkville, VIC 3052 Australia; Department of Medicine, The University of Melbourne, Parkville, VIC 3010 Australia; Department of Medical Biology, The University of Melbourne, Parkville, VIC 3010 Australia

## Abstract

Despite advances in the treatment of patients with early and metastatic breast cancer, mortality remains high due to intrinsic or acquired resistance to therapy. Increased understanding of the genomic landscape through massively parallel sequencing has revealed somatic mutations common to specific subtypes of breast cancer, provided new prognostic and predictive markers, and highlighted potential therapeutic targets. Evaluating new targets using established cell lines is limited by the inexact correlation between responsiveness observed in cell lines versus that elicited in the patient. Patient-derived xenografts (PDXs) generated from fresh tumor specimens recapitulate the diversity of breast cancer and reflect histopathology, tumor behavior, and the metastatic properties of the original tumor. The high degree of genomic preservation evident across primary tumors and their matching PDXs over serial passaging validate them as important preclinical tools. Indeed, there is accumulating evidence that PDXs can recapitulate treatment responses of the parental tumor. The finding that tumor engraftment is an independent and poor prognostic indicator of patient outcome represents the first step towards personalized medicine. Here we review the utility of breast cancer PDX models to study the clonal evolution of tumors and to evaluate novel therapies and drug resistance.

## Introduction

Breast cancer is not a single disease but a diverse set of diseases characterized by heterogeneity in histology, genomic aberrations, and protein expression that influence treatment response and patient outcome. Importantly, this heterogeneity cannot be precisely defined through the traditional parameters of histopathology, tumor size, grade, nodal involvement, and biomarker expression that are currently used to guide treatment decisions. Although survival rates following diagnosis have improved in recent years, patients with recurrent disease are almost invariably treatment resistant, highlighting the need for identifying new therapeutic strategies.

The heterogeneity of breast cancer is a significant stumbling block for the application of personalized medicine approaches. For this strategy to be successful, a complete set of clinically relevant and validated biomarkers is required, along with the development of companion diagnostic tests to evaluate treatment responses [1]. To date, these platforms do not exist for breast cancer. Nevertheless, a more refined breast cancer classification system has been developed over the past 15 years, integrating information based on gene expression arrays. Five intrinsic clusters were initially defined – luminal A, luminal B, basal-like, human epidermal growth factor 2 (HER2) over-expressing, and the normal breast-like subtypes. The precise characteristics of the latter group remains unclear. These subtypes can predict clinical behavior including overall survival, patterns of metastasis, and response to treatment [2-5]. More recently, other subtypes have been defined, notably the claudin-low tumors, which are predominantly triple-negative and exhibit mesenchymal features [2] and a stem cell-like expression signature [2,6]. The different tumor subtypes are likely to result from distinct cells of origin, unique differentiation blockades, and different repertoires of mutations [7]. It is essential to decipher the molecular and cellular differences amongst the subtypes in order to develop a personalized medicine approach.

Over recent years, patient-derived xenograft (PDX) models have emerged as important tools for translational research, with the promise of enabling a more personalized approach to patient care. In this review, we discuss the importance of these models for assessing novel therapies and understanding molecular and cellular mechanisms that contribute to tumor evolution.

## Inter-tumoral heterogeneity in breast cancer

The traditional histopathological markers used in the clinic do not always reflect the intrinsic subtype [5]. For example, ~10% of basal-like tumors and 15 to 20% of claudin-low tumors are hormone receptor-positive at the mRNA level [8]. Surrogate immunohistochemical markers have been suggested, including cytokeratin 5/6 and epidermal growth factor receptor for basal-like tumors [9], and proliferative indices such as Ki67, which may demarcate luminal B from luminal A tumors [5,10]. Indeed, proliferation markers are heavily weighted in current recurrence risk scores including the Oncotype DX Test (Genomic Health, Redwood City, CA, USA) [11]. These data are prognostic and provide clinicians with information to aid decision-making, particularly with respect to those patients who would derive little benefit from chemotherapy, thereby sparing them from its potential toxicity. Despite this improved molecular classification, differences remain within each intrinsic subgroup, perhaps reflecting the activation or inactivation of different signaling pathways, and differing cell–cell and cell–matrix interactions within the tumor microenvironment. Although multigene expression assays (either arrays or RNA-seq) are useful, mutational analysis may provide higher gain as a mutation can imply causality [12]. Ultimately, an integrated multiplatform analysis that encompasses genomics, transcriptomics, and proteomics will likely be required.

All cancers carry somatic mutations due to imprecise repair of DNA damage. Mutations may be single base-pair substitutions or structural variants including translocations, large deletions, and intra-chromosomal inversions (reviewed in [12]). Only a small proportion of these mutations are considered to be driver mutations that promote tumorigenesis [13]. Other mutations are referred to as passenger mutations that contribute little to the malignant phenotype. Large-scale parallel sequencing has revealed subtype-associated gene mutations as well as a small number of genes that are frequently mutated across multiple breast cancer subtypes such as TP53 and PIK3CA/PTEN [14]. Interestingly, the overall mutation rate was found to be lowest in luminal A cancers relative to the basal-like and HER2 subtypes. In one of the largest studies assessing 2,000 breast tumors representing all major subtypes with copy number alteration and gene expression analyses, 10 novel subclassifications of breast cancer were proposed [15]. These subclassifications overlap with the intrinsic breast cancer subtypes and revealed further heterogeneity within the estrogen receptor (ER)-positive subgroup, differing in copy number and *cis*-acting alterations. In other large cohorts, recurrent mutations in genes not previously associated with breast cancer (for example, *TBX3*, *MLL3*, *RUNX1* and *CBFB*) and novel translocations were identified [16,17].

With respect to clonal heterogeneity in breast cancer, genomic analysis of triple-negative breast cancers has indicated that some are characterized by a few dominant clones, whereas others may comprise more than 15 [18]. Whole genome sequencing of a primary lobular breast cancer and a metastasis occurring in the patient diagnosed 9 years later [18] showed that only 11 of 32 mutations found in the primary tumor were detectable in the metastatic lesion, indicating significant genetic evolution during the metastatic process. This study also suggested that the majority of heterogeneity occurs within, and not across, the different breast cancer subtypes.

## Derivation of patient-derived xenograft models

The use of preclinical models to test hypotheses is central to cancer research. Unfortunately, long-established human cell lines, and many transgenic mouse models, often fail to recapitulate the key aspects of human malignancies and thus do not adequately predict drug effects in the clinic. Cancer cell lines have been used since the 1970s as an *in vitro* model for drug discovery. Whilst they serve as useful tools, there are significant limitations, because continual passage of these cell lines is accompanied by extensive clonal selection and consequent loss of heterogeneity [19,20]. Moreover, different isolates of the same cell line can differ from one another at both the genomic and gene expression levels [21]. Their lack of predictive value is highlighted by the absence of correlation between clinical results and *in vitro* and *in vivo* data obtained with cell lines [22], in part contributing to the >90% failure rate for the development of new oncology drugs [1]. Indeed, transcriptome studies of clinical samples versus established cancer cell lines showed that cell lines were more closely aligned to each other, regardless of the tissue of origin, than to the clinical samples they were intended to model [19].

In contrast to long-established cell lines, PDXs are propagated through successive generations in murine hosts, thus circumventing the high selection pressure imposed by *in vitro* culture. Multiple groups have now established cohorts of breast cancer PDXs [23-32]. Importantly, like cell lines, PDXs provide a renewable tissue resource. Overall, PDXs recapitulate breast cancer-specific gene expression patterns of primary tumors, exhibit stable patterns of protein expression, and have relatively stable genomes over time (see 'Using patient-derived xenografts to interrogate clonal evolution and metastasis').

## Varying parameters in the generation of patient-derived xenograft models

**Figure 1:**
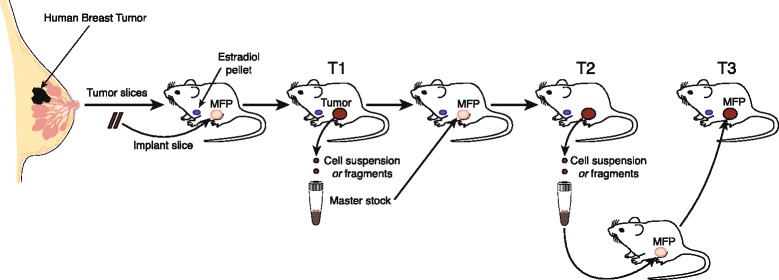
**Derivation of patient-derived xenograft models of human breast cancer in mice.** Tumor slices are implanted into the cleared inguinal fat pad of mice (MFP) concurrently with a subcutaneous estradiol pellet. Following growth of the tumor in passage 1 (T1), a single-cell suspension or tumor fragments can be prepared for sequential passage of the tumor. It is advisable to prepare frozen aliquots of cell suspensions or tumor fragments at T1 and T2, as a source of early passage tumor.

The methodology underpinning the generation of human breast cancer xenograft models has been comprehensively described [33,34]. Briefly, for the initial transplantation, tumors derived from primary surgical resection are sliced into fragments, and then implanted into immunocompromized mice. In other cases, cell suspensions have been derived from pleural or peritoneal fluid for injection. For tumor passage, either fragments or tumor cell suspensions may be utilized to maintain the PDX line (Figure 1). It is of paramount importance to freeze early passage tumors (after the first and second passages) as viable samples in order to create a live bank of early passage tumor cells for experimental studies. Although tumor fragments have been readily engrafted [33,34], one potential advantage of using cell suspensions derived from a frozen vial is that they allow inoculation of mice in any given cohort with the same number of tumor cells [30,35]. Over the past two decades, considerable progress has been made in improving the take rates of breast cancer xenografts. This progress has included implantation into the orthotopic site, estrogen supplementation, the use of more highly immunosuppressed mice, as well as altering the microenvironment through the addition of mesenchymal stem cells [25] and/or Matrigel [36,37].

Historically, breast tumors were often implanted subcutaneously, but orthotopic implantation into the inguinal mammary fat pad is optimal because this more faithfully recapitulates the breast tumor stromal microenvironment [25,31,38]. The stroma comprises the vasculature, adipocytes [39], tumor-associated macrophages and other immune cells [40], as well as cancer-associated fibroblasts [41], all of which secrete growth factors/cytokines that influence tumor cell behavior in a paracrine or juxtacrine fashion. However, not all murine growth factors and cytokines interact with their human counterpart receptors, which may substantially affect the tumorigenesis process. Vascularization of orthotopic tumors was noted to be significantly higher than that of subcutaneous tumors, and enhanced engraftment rates were observed by implantation into the inguinal rather than thoracic fat pads, highlighting the impact of the local microenvironment [36].

Table 1 summarizes the current data for PDX models using the orthotopic site and underscores the bias towards engraftment of higher grade, triple-negative or luminal B tumors derived from primary tumor resections or metastatic effusions. It is noteworthy that orthotopic models of pancreatic carcinoma have been found to more accurately predict a patient’s response to chemotherapy than those implanted into a heterologous site [42]. Curiously, implantation into the renal capsule has been reported to increase engraftment rates (for example, for the lung [43]) and shorten the time to engraftment, independent of tumor origin, although a comparison has not yet been performed in the case of breast cancer.

**Table 1:** **Generation of orthotopic patient-derived xenografts from primary breast cancer and metastatic tissue**

**Study**	**Mouse strain**	**Supportive conditions**	**Stable take rate**	**Tissue source**	**IHC subtypes of PDX**	**Correlation with engraftment**	**PDX concordance with source tissue**	**Metastases**
Al-Hajj and colleagues [23], Liu and colleagues [76]	NOD/SCID	Estradiol	NR	Primary breast (6)	4 TNC	NR	NR	5/8 (62%) micrometastases
	NSG	Etoposide i.p.		Pleural effusion (2)	2 HER2^+^			
		Matrigel			2 ER^+^			
Fleming and colleagues [36]	NOD/SCID	Estradiol	NR	TNC pleural effusion (2)	NR	Increased engraftment in abdominal versus thoracic mammary gland	NR	NR
		Etoposide i.p.						
		Matrigel						
DeRose and colleagues [25]	NOD/SCID	Estradiol	12/49 (27%)	Primary breast (4)	5 TNC	Similar engraftment for primary and metastatic tumors	Histological PAM 50 expression profiling	10/12 to lymph node, lung and peritoneum
				Pleural effusion (7)	2 HER2^+^	TNC grew fastest	Genomic	
				Ascites (1)	3 ER^+^/HER2^+^	Increased tumor growth with serial passage		
					2 ER^+^	Engraftment as a prognostic indicator of disease outcome		
Vaillant and colleagues [30], Oakes and colleagues [35]	NSG	Estradiol	37/158 (23%)	Primary breast (37)	17 TNC	TNC and HER2^+^ higher engraftment	Histological	NR
					13 ER^+^			
					2 ER^−^PR^+^			
					5 HER2^+^			
Ma and colleagues [58]	NOD/SCID	Fibroblasts^a^	NR	Primary breast (1)	3 TNC	NR	PAM 50 expression profiling	NR
				Ovarian metastasis (1)			Genomic	
				Brain metastasis (1)				
Kabos and colleagues [37]	NOD/SCID	Estradiol		Primary breast (6)	2 TNC	Nonluminal higher take rate than luminal tumors	Histological	NR
	NSG	Matrigel	10/24 (42%)	Metastatic effusion (2)	8 ER^+^			
Zhang and colleagues [32]	SCID/Bg	Estradiol	6/32 (19%) in NSG mice, 1/38 SCID/Bg (no E2), 1/29 SCID/Bg (E2 + fibroblasts), 15/70 (21%) SCID/Bg (E2)	Primary breast (22)	12 TNC	TNC and grade III have higher take rate	Histological	12/25 (48%) lung metastases
	NSG	Fibroblasts^a^		Ascites (2)	3 HER2^+^		Clinical response	
				Pleural effusion (1)	2 ER^+^			
Li and colleagues [49]	NOD/SCID	Fibroblasts^a^	22/152 (13%)	Primary breast	12 ER^/^HER2^−^	NR	Histological	NR
				Nodal metastasis	2 HER2^+^		Immunohistochemical	
				Skin metastases/recurrence	7 ER^+^/HER2^−^		Proteomic	
					1 ER^+^/HER^**+**^		Genomic	
Zhang and colleagues [71]	NOD/SCID	Matrigel	NR	Primary breast (6)	7 TNC	NR	Histological	NR
				Soft tissue metastasis (1)			Biomarker expression	
							PIKC3A sequence	
							Genomic	

Bg, beige; E2, estradiol; ER, estrogen receptor; HER2, human epidermal growth factor receptor-2; IHC, immunohistochemistry; i.p., intraperitoneally; NSG, NOD/SCID/IL2γ-receptor null; NOD, nonobese diabetic; NR, not reported; PDX, patient-derived xenograft; PR, progesterone receptor; SCID, severe combined immunodeficiency; TNC, triple-negative cancer. ^a^Irradiated and unirradiated for humanization.

Given the importance of the tumor microenvironment, different groups have attempted to humanize the mouse mammary fat pad. Based on increased engraftment of human mammary epithelial cells in nonobese diabetic (NOD)/ severe combined immunodeficiency (SCID) mice by co-introduction of an immortalized human fibroblast cell line [44], Zhang and colleagues explored a number of variables and paradoxically found that the introduction of immortalized human fibroblasts derived from normal tissue was inhibitory rather than stimulatory to xenograft growth [32]. In another study, co-engraftment of primary human mesenchymal stem cells showed that they contributed to maintaining phenotypic stability of the tumors and their vascular density, as well as reducing necrosis [34]. Exogenous estrogen stimulates growth of breast cancer PDXs and is critical for engraftment; for example, estrogen improves take rates in SCID/Beige mice from 2.4% in the absence of supplementation to 25% in its presence [32]. Interestingly, both ER-positive and ER-negative tumors benefit from estrogen supplementation [32]. In ER-negative models, this is presumably through ERα-mediated stimulation of bone marrow-derived myeloid cells that promote angiogenesis and tumor growth [45].

Multiple mouse strains are available, with slight differences in immunosuppression conferring both advantages and disadvantages. For melanoma, the level of immunosuppression in recipient mice is a key determinant of xenograft establishment [46]. NOD/SCID/IL2Rγc^−/−^ (NSG) mice are more immunosuppressed and demonstrate higher engraftment rates. In breast cancer, however, NSG and SCID/Beige mice appear to have similar take rates [32], while the NSG strain is superior to NOD/SCID (GJL, JEV unpublished data).

## Validation of patient-derived xenograft models for preclinical research

All clinically defined subtypes of breast cancer have been established as PDX models [25,28,32,37], with a bias towards triple-negative tumors owing to their higher rates of engraftment. In addition, samples from younger or node-positive patients show increased engraftment [32]. ER-positive PDX models have been historically difficult to grow, and those that engraft represent luminal B (rather than luminal A) tumors that are characterized by high Ki67 scores [25,37]. These ER-positive tumors retain their hormone receptor status over multiple passages and their dependence on estrogen for growth. Most emphasis in the field has been on engraftment of primary tumors, although pleural effusions and occasional metastases have been used for establishing PDX lines. It is important to note, however, that these are likely to be distinct from the primary tumor, which remains the preferred source of engrafting material in order to study the different stages of tumorigenesis. An important unmet need is the generation of paired primary and metastatic breast cancers.

Early passage breast PDX models have been shown to retain the principal molecular characteristics of the corresponding patient tumor at both the genomic and gene expression levels [25,32]. These appear to be relatively stable during sequential passage over several generations. Although the gene expression profiles of triple-negative breast cancer PDXs recapitulate that of the parent tumor, passaging can lead to the emergence of a more aggressive type with a higher proliferative index [28]. Profiling of breast PDXs grown in Swiss nude mice established that less than 5% of genes varied from the parental tumor and that the majority of these were human stroma specific [29]. This probably arises upon adaptation of the tumor to the mouse microenvironment. Likewise, comparison of patient tumors with their counterpart PDX has revealed variation in the expression of human extracellular matrix proteins [47]. Metabolic profiles (such as for choline) of breast PDX models also showed concordance with their corresponding patient samples [48].

Genome-wide analyses have revealed that structural and copy number variations are faithfully retained in a large bank of PDXs and their originating tumors [8,49]. Variant allele frequencies were largely preserved in the PDXs, highlighting the transplantability of clonal heterogeneity [49]. In an independent analysis of 20 breast PDXs, 25% of which were ER-positive, the PDX models also recapitulated the expression and copy number alterations of the parent tumors [50]. Although the vast majority of copy number alteration profiles were found to overlap, a small number of copy number alterations were lost on serial passaging. The PDX tumor may stabilize around a dominant clone, particularly on extended passage. At the single nucleotide level, a small number of single nucleotide variants were found to be PDX unique, but most were expressed below the detection limit, suggesting they are nonfunctional, passenger mutations [49]. These nonfunctional mutations may have arisen during sequential passaging or possibly pre-existed in the primary tumor at a previously undetectable level.

Because multiple passages may be required to expand the model for therapeutic use, Zhang and colleagues performed gene expression analysis on every fifth generation, and demonstrated that generations clustered together across at least five generations and up to 15 generations [32]. Moreover, reverse phase protein assay expression analysis and single nucleotide polymorphism analysis confirmed that the xenograft models were stable at the genomic, protein and phospho-protein levels [32]. Nevertheless, it seems prudent to utilize early passage PDX for preclinical studies to circumvent inevitable clonal selection and to verify the integrity of the xenografted tumors at the molecular level.

## Using patient-derived xenografts to interrogate clonal evolution and metastasis

Tumors are recognized to comprise multiple subclones, based on the analysis of samples from an individual patient at different times [18], and hypermutable loci [51]. In leukemia, single-cell isolation has provided evidence for complex evolutionary mechanisms, including a nonlinear branching model of tumor evolution [52]. In the context of breast cancer, deep genomic analyses of a basal-like primary tumor and brain metastasis from a patient, and of a PDX model derived from the primary tumor, showed that the metastatic lesion contained *de novo* mutations and deletions not present within the primary tumor. While the xenograft retained the primary tumor mutations, it most closely mimicked the mutation spectrum of the metastasis; that is, the PDX was genetically closer to the metastatic lesion than the primary tumor [53]. In another study, comparison of PDXs at different time points detected multiple single nucleotide variants, but these mostly localized within the noncoding regions, suggesting that they arose over time during passaging [49].

PDX models have been demonstrated to undergo metastasis in the mouse but it is unclear whether this accurately recapitulates metastases observed in patients. In the case of three primary tumors capable of metastasis in mice, although these correlated with the clinical metastases, additional sites were apparent [25]. For example, whereas only lymph node metastases were apparent in patients, both lymph node and lung metastases were observed in the corresponding mouse PDX model. Moreover, while PDXs derived from pleural effusions in part reflected metastases observed in patients, there was a predilection for lung and lymph node metastases in mice. In a separate PDX cohort, lung metastases were seen in 12 strains of mice, but were not evident in the patients from which they were derived [32]. Interestingly, the presence of lung metastases in these mice was strongly associated with the detection of circulating tumor cells [54]. Although brain metastases occurred in some patients [32], it remains to be determined whether this can be recapitulated in mouse PDX models.

## Preclinical patient-derived xenograft models for therapeutic studies

**Figure 2:**
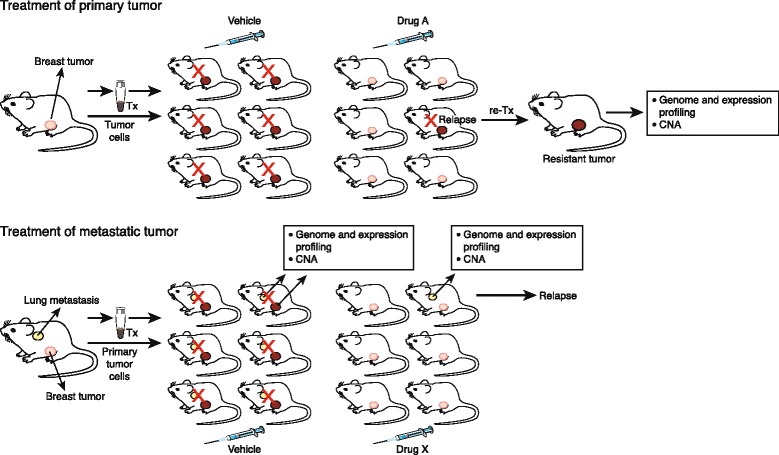
**Evaluation of response of primary tumor and its metastases using patient-derived xenograft models.** Upper panel: A cohort of immunocompromised mice are divided into two arms and treated with either vehicle or drug A. A relapsed tumor can be passaged in mice to generate a resistant tumor line for genomic and gene expression analyses. Lower panel: Genomic and expression profiling of the primary tumor and metastasis (for example, the lung) may identify potential therapeutic targets for metastasis. In the case shown, drug X eradicated the primary tumor but not all metastases. CNA, copy number alterations; Tx, treatment.

One of the major issues in drug development is the absence of correlation between preclinical data and trial results leading to failure of multiple phase III studies, in part due to the poor predictive value of cell line studies [19,20]. PDX models are being increasingly used for drug discovery and development, with the potential for further understanding tumor progression and metastases, and their eradication through specific targeting (Figure 2).

### Basal-like patient-derived xenograft models

Basal-like tumors represent ~20% of patients with breast cancer, are typically triple-negative, and carry a worse prognosis due to high rates of local and systemic relapse. The absence of ER and HER2 expression precludes the use of endocrine therapy or anti-HER2 treatment. Systemic treatment is limited to cytotoxic chemotherapy, thus highlighting the need for biomarker development and new treatment strategies [55]. Human-in-mouse PDX models have established the biological rationale for new classes of treatment, including inhibitors of the phosphatidylinositol 3-kinase (PI3K) pathway [56,57], checkpoint kinase 1 [58], Aurora kinase [59], inactivation of NOTCH signaling [60] by gamma secretase inhibitors or a neutralizing antibody [61], or WNT pathway ablation [62]. Moreover, these agents were found to enhance the efficacy of chemotherapy in the preclinical studies. Triple-negative breast cancer shows relatively fewer somatic mutations but a high degree of genomic rearrangement [63], suggesting that DNA-damaging agents may be efficacious in the treatment of these tumors.

Impairment of apoptosis is a hallmark of cancer [64], thus generating intense interest in the BCL-2 family of proteins for the development of a new class of agents termed BH3 mimetics. BCL-2 is overexpressed in approximately 75% of breast cancer – including 83% of luminal tumors, 50% of HER2-positive tumors and 18.5% of basal-like tumors [35] – and has emerged as an important prognostic marker of luminal tumors [65]. ABT-737, which targets BCL-2, BCL-XL, and BCL-W [66], was found to potentiate the response to docetaxel in PDX models of triple-negative breast cancer [35], suggesting that elevated BCL-2 expression in this subtype constitutes a predictive marker.

### Estrogen receptor-positive patient-derived xenograft models

Most ER-positive PDX models remain dependent on estrogen for tumor growth [32,34,37], and respond to endocrine therapy with features consistent with clinical responses of the tumor of origin. In ER-positive PDX models, ABT-737 or the specific BCL-2 inhibitor ABT-199 markedly improved responsiveness to tamoxifen [30]. These data indicated that targeting of BCL2 (and not Bcl-X or BCL-W) was essential for inhibition of tumor growth. Of note, in one PDX line (23 T), the combination of ABT-199 with tamoxifen resulted in complete remission. Interestingly, in another ER-positive model displaying only a partial response, higher levels of P-AKT were apparent and synergistic activity was observed between ABT-199, tamoxifen and mammalian target of rapamycin (mTOR) inhibition [30]. Recent analysis of ER-positive PDXs has provided insight into novel estrogen-dependent signaling pathways [37].

### HER2-positive patient-derived xenograft models

Very few therapeutic studies have thus far been carried out with this type of PDX model. However, Marangoni and colleagues tested two HER2/ERBB2-amplified PDX lines, one of which responded to trastuzumab while the other did not [27]. Synergistic interactions of trastuzumab with docetaxel were reported to increase anti-tumor efficacy. Inhibitors of the PI3K/Akt/mTOR pathway in combination with anti-HER2 therapies also have been evaluated. A PDX model derived from a patient who had relapsed after trastuzumab therapy was unresponsive to trastuzumab, but showed tumor shrinkage in response to lapatinib and superior regression with a combination of lapatinib and the mTORC1/2 inhibitor INK-128 [67].

## Patient-derived xenograft models of drug-resistance

Although the majority of ER-positive breast cancer diagnoses respond to anti-estrogens such as tamoxifen and aromatase inhibitors, their efficacy is limited by intrinsic and acquired resistance. Luminal PDX models with acquired *in vivo* endocrine resistance have been recently generated and identified significant deregulation of ER-mediated gene transcription, suggesting that endocrine resistance is both tumor specific and treatment specific [68]. Tamoxifen-resistant tumors were generally resistant to endocrine therapy; however, xenografts with acquired resistance to estrogen deprivation retained some sensitivity to tamoxifen. Resistance to tamoxifen did not produce a universal molecular phenotype of endocrine resistance, but rather a diversity of endocrine-resistance phenotypes. The data from these PDX models are consistent with clinical observations that patients who progress on one form of endocrine therapy may still derive clinical benefit from another, and highlight the different forms of endocrine resistance that probably occur in patients. Li and colleagues recently invoked inter-tumoral heterogeneity to explain *de novo* endocrine-therapy resistance in ER-positive breast cancer and discovered point mutations or rearrangements affecting the *ESR1* ligand-binding domain, which were unique to the different tumors and were not previously identified by cell line studies [49]. These findings suggest that functional *ESR1* variants may be selected in a subset of endocrine-resistant luminal tumors and have immense potential to inform the design of effective therapies to target mutant forms of ESR1. Other mechanisms of endocrine resistance, largely based on cell lines and cell line xenografts, include the utilization of alternate signaling pathways (such as PI3K/AKT/mTOR or human epidermal growth factor B2 receptor/HER2), increased AP1 expression, deregulation of ER-associated co-regulators, or deregulation of the cell cycle and apoptosis [69].

Indeed, aberrant PI3K signaling has been implicated as a potential mechanism of endocrine resistance, with upregulation of AKT signaling and acquired sensitivity to mTOR inhibition. The mTOR inhibitor everolimus has been beneficial in the treatment of advanced breast cancers resistant to endocrine therapies [70]. In tamoxifen-resistant xenografts, treatment with everolimus alone or in combination with tamoxifen resulted in a cytostatic response; in contrast to combined fulvestrant and everolimus, which resulted in significant regression [68]. Increased activity of the PI3K pathway is also associated with basal-like breast cancers. The treatment of a panel of PDXs representing multiple triple-negative subtypes with mTOR inhibitors led to significant tumor growth inhibition but no tumor was eradicated, indicating the need for testing combinational therapies in future investigations [71]. Resistance to mTOR inhibition has been proposed to reflect AKT activation through a negative feedback loop. This has been investigated in PDX models of basal-like breast cancer using combined mTOR and AKT inhibitors. Synergy was demonstrated, with an even more dramatic reduction in cell proliferation and tumor growth following knockdown of PTEN [56]. Given the prevalence of PTEN-inactivating mutations in patients with basal-like breast cancer, this therapeutic approach could be explored further.

PDX models are showing increasing utility for the identification of mechanisms of resistance and potential targetable pathways. For example, reverse phase protein assay analysis in combination with an integrated bioinformatic model established upregulation of the PI3K/Akt/mTOR signaling pathway as a candidate driver of resistance to anti-angiogenic agents [72]. Moreover, PDX models can be used to create resistance models for preclinical research. Prolonged exposure to cisplatin in high-grade serous ovarian cancer has led to the generation of platinum-resistant models [73], while continuous vemurafenib treatment led to the development of a resistant BRAF-mutated melanoma PDX model and the identification of a new treatment strategy [74]. In the case of breast cancer, continuous treatment of HER2-amplified PDX models with trastuzumab could provide a useful preclinical tool to eventually overcome HER2 resistance through the identification of culprit pathways.

## Clinical correlates and patient-derived xenograft models

**Figure 3:**
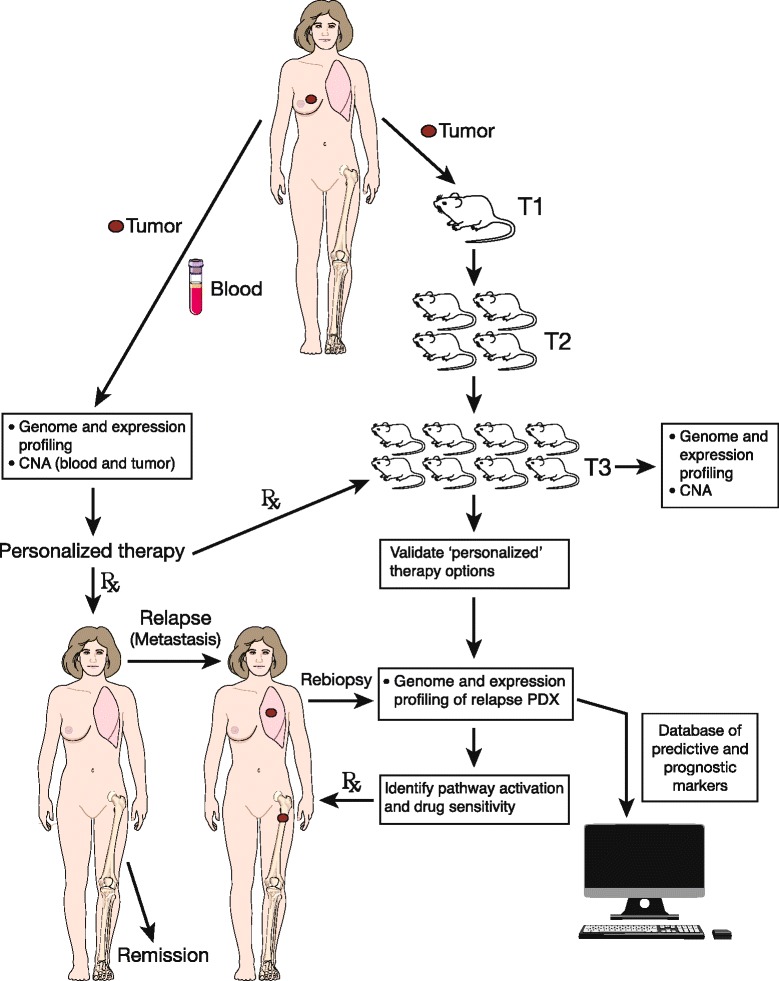
**Idealized personalized medicine strategy integrating data from mouse patient-derived xenograft models with patient treatment.** Rather than directly assigning breast cancer patients to standard therapy, patients are treated on the basis of genomic and gene expression analyses. Blood from the patient is used as a reference for copy number alteration (CNA) analysis. In parallel, tumor fragments are xenografted into mice to establish a patient-derived xenograft (PDX) model. The patient tumor and their corresponding PDX tumor at transplant 3 (T3) undergo comparative genomic and gene expression analyses. Mice are treated with inhibitors (chemotherapy, antibodies or small molecules) based on initial genetic analysis of the patient tumor, in order to identify/validate the agents to be used for clinical treatment and to identify refractory tumors. If relapse occurs, re-biopsy and analysis of the metastatic/recurrent tumor together with the refractory PDX model could be used to reveal pathway activation. A database of mutations, expression profiles, and tumor response from multiple patients can be created to guide therapy for future patients. Rx, treatment of patient.

The most relevant data on the power of PDXs will ultimately derive from direct comparison of a patient’s clinical response with that of the corresponding PDX to the same drug (Figure 3). Although limited in number, similarities between patient outcome and PDX responses have been reported. High concordance was observed between the clinical response and the corresponding xenograft model in 12 out of 13 cases, although these represented resistance to therapy [32]. In another study, five of seven xenografts predominantly from high-grade, triple-negative tumors demonstrated a concordant response [27]. Similar observations have been made for other solid malignancies including high-grade serous ovarian cancer for platinum sensitivity [75].

## Patient-derived xenograft models for breast cancer stem cell characterization

The cancer stem cell hypothesis provides a cellular mechanism to account for phenotypic and functional intra-tumoral heterogeneity. Furthermore, it provides an explanation for resistance to radiation and chemotherapy, as well as eventual tumor relapse. A number of potential markers of breast cancer stem cells have been identified (CD44^+^CD24^−^, ALDH1^+^), but these do not universally mark breast cancer stem cells, with variation evident between individual tumors. In cases where tumor samples are too small for cell fractionation, early passage xenografts may be a useful tool for evaluating the existence of cancer stem cells and determining their intrinsic resistance to therapy [46]. It has been speculated that some ER-positive tumors may have luminal-type cancer stem cells that are yet to be discovered [37]. PDX models transduced with a reporter to enable tracking of cells have indicated that CD44^+^ breast tumor cells enriched for cancer stem cells spontaneously metastasize to the lungs and lymph nodes, thus suggesting a role for cancer stem cells in primary tumor growth as well as metastatic spread [76]. Further definition of the role of cancer stem cells through xenotransplantation studies may provide strategies that target both cancer stem cells and non-cancer stem cells, which is ultimately required for achieving durable response and remission.

## Pitfalls associated with patient-derived xenograft models

Despite the ability of PDX models to recapitulate the primary tumor, there are several limitations of which researchers need to be cognizant. To date, the majority of PDX cohorts are biased towards more aggressive tumors, characterized by low ER-positivity, high Ki67, and node positivity. Indeed, the rate of engraftment may serve as an independent predictor of poor patient outcome [34]. Samples from metastatic lesions demonstrate an improved take rate and growth, but do not allow the study of naïve tumor tissue. Therefore, rates of engraftment skew the intrinsic subtype representation of PDX models and do not fully encompass inter-tumor heterogeneity, and preclinical results using aggressive tumors may not be completely relevant to lower grade tumors. Moreover, in some cases, the signature of the PDX may be more concordant with metastatic lesions than the primary tumor itself, suggesting that selection occurs within the initial passaging of the xenograft [42,53]. In these cases, genomic rearrangements may reflect an intrinsic metastatic potential. One other consideration is the propensity for viral contamination of PDX lines, particularly with lactate dehydrogenase-elevating virus. Although this has proven difficult to eradicate, it nevertheless seems possible based on a recent report [76]. Use of lactate dehydrogenase-elevating virus-contaminated Matrigel in early studies may account for some instances of contamination, and some groups have produced their own Matrigel in order to avoid potential viral contamination [34]. Other potential sources of this virus may include bedding or food sources.

Several parameters require optimization to more closely mimic the genesis of human tumors. Given that the stromal compartment and immune system play an important role in breast cancer progression [77], the loss of human stroma following engraftment is problematic. The rapid replacement with murine stroma [25] may result in changes to paracrine regulation of the tumor and its biological properties [78], owing to species-specific differences in ligands and/or receptors. Moreover, immune cells are critical for breast tumorigenesis and metastasis. One of the major limitations of xenotransplantation is the necessity for immunocompromised mice in order to allow tumor engraftment. Immunocompromised strains such as NSG mice lack natural killer cells, and both B and T lymphoid cells, thus precluding PDX models for the preclinical testing of immunotherapies in breast cancer. Rather, syngeneic mice must be utilized for this purpose. Humanization of the mouse immune system by co-engraftment of human bone marrow cells may circumvent some of these issues but this introduces an extra layer of complexity [79]. Notably, a genetically engineered prolactin-humanized mouse expressing physiological levels of human prolactin demonstrated a greatly improved take rate for ER-positive luminal breast cancers, which were found to be more responsive to tamoxifen [80]. The targeted knockin of multiple human cytokine genes into their respective loci within mice would be anticipated to further improve tumor engraftment.

## Conclusions

The wealth of data derived from massive parallel sequencing has now primed the genomic phase of clinical trials with the potential integration of predictive and prognostic biomarkers (Figure 3). Through the development of mouse clinical trials, it may be feasible to predict more relevant clinical treatments and to optimize novel therapeutics more rapidly than in a strictly clinical setting, and in a cost-effective manner. However, the prospect of generating individual PDX avatars based on concordance between patient and PDX responses is challenged by low engraftment rates and the time required (several months) to establish a PDX model, as well as the significant costs associated with maintenance of mouse colonies. In order for PDXs to be relevant at the level of the individual patient and to integrate drug screening, the engraftment rate – particularly of ER-positive and HER2-positive tumors – needs to be radically increased, and the time required for engraftment needs to be radically reduced, without compromising biological fidelity relative to the tumor of origin. Early biopsy and engraftment of samples might eventually allow determination of important changes in the tumor at the time when tumor resistance becomes clinically apparent.

Given the current clinical challenge of eradicating metastatic disease, there is a pressing need for models that better predict metastatic behavior. Xenograft lines with metastatic capacity to the lung, node, and pleura, but not to the liver or bone as yet, have been described [25,32]. PDX models expressing reporter genes have proven instrumental in the tracking of these to form spontaneous metastases in the lung and distant nodes [76]. Although orthotopic transplantation may recapitulate metastasis observed in patients, it does not always correlate. Nonetheless, if a similar metastatic pattern can be generated in PDX models, then the inclusion of a fluorescent or luminescent marker may allow the earliest molecular and cellular events in metastatic dissemination *in vivo* to be studied and the eventual identification of therapies to target metastases.

## Abbreviations

ER: Estrogen receptor
HER2: Human epidermal growth factor 2
mTOR: Mammalian target of rapamycin
NOD: Nonobese diabetic
NSG: NOD/SCID/IL2Rγc^−/−^
PDX: Patient-derived xenograft
PI3K: Phosphatidylinositol 3-kinase
SCID: Severe combined immunodeficiency
